# ANRGs impact on gastric cancer progression and drug efficacy: A comprehensive study

**DOI:** 10.1097/MD.0000000000034861

**Published:** 2023-10-27

**Authors:** Zhijing Zhang, Yeqing Zhu

**Affiliations:** a Pharmacy, Fuyang Hospital Affiliated to Anhui Medical University, Fuyang, China.

**Keywords:** chemotherapeutic drug sensitivity, GC prognosis, immune microenvironment, small molecule compounds, TCGA

## Abstract

Gastric cancer (GC) is a significant contributor to cancer-related mortality globally, with the heterogeneity of metastasis and treatment impacting patient prognosis. Currently, the treatment of GC still relies on early surgical resection, and comprehensive treatment is needed for patients with metastatic GC. Anikis-related genes (ANRGs) have been shown to affect tumor metastasis. Exploring the role of ANRGs in GC will help us understand the mechanism of tumor metastasis; screening precise targets and selecting appropriate chemotherapeutics will help individualize the treatment of GC patients. In this study, we established a prognostic scoring model based on ANRGs and explored their association with GC patient prognosis, immune microenvironment, chemotherapeutic drug sensitivity, and small molecule compounds. Our findings revealed that a gene signature composed of ANXA5, CCN1, EGF, VTN, and ZBTB7A accurately predicted GC patient prognosis. Patients in the low-risk group had better outcomes, higher macrophage M1 infiltration, and higher tumor mutation burden. The half maximal inhibitory concentration (IC50) values of Ponatinib (ap.24534), Motesanib (amg.706), and Navitoclax (abt.263) were lower in the high-risk group, indicating that patients in the high-risk group were more sensitive to these chemotherapy drugs, meaning with better clinical outcomes. In addition, we screened the small molecule compound SGC-CBP30 that can inhibit ANXA5 and CCN1, and these results help individualized treatment of GC patients. Our study identified key genes based on ANRGs and developed a novel gene signature for predicting the prognosis of GC patients and understanding the relationship between immunity and tumor mutation burden. Additionally, we identified chemotherapeutic drugs that can guide GC treatment and elucidated the binding affinity between specific targeted drugs and distinct protein sites, providing novel insights for the precise treatment of GC patients.

## 1. Introduction

As the world’s third leading cause of cancer death, more than 1 million people are diagnosed with gastric cancer (GC) every year.^[1]^ Because patients with early-stage GC are usually asymptomatic, most patients with GC are detected at an advanced stage.^[2]^ The 5-year survival rate of patients with early GC is >95 percent, while the 5-year survival rate of patients with advanced GC is <5 percent.^[3]^ GC patients have evident heterogeneity in different treatments, which has a significant impact on the prognosis of patients.^[4]^ Despite advances in chemotherapy regimens for advanced GC, the chemotherapy effect is still unsatisfactory, and the overall survival (OS) is less than 2 years.^[5,6]^ Targeted therapy for GC is the direction to prolong the survival time of patients, but many targeted drugs have not achieved the expected results.^[7]^

In the absence of extracellular matrix attachment or when adhered to inappropriate sites, cells undergo specially programmed death, called anoikis,^[8,9]^ which is one of the methods to prevent tumor cell metastasis. Correspondingly, cunning tumor cells also avoid anoikis by secreting growth factors, activating pro-survival signaling pathways, or changing the expression pattern of cellular integrins.^[10]^ Studies in recent years have shown that resistance to anoikis is gradually accepted as a marker of tumor cells and is involved in the process of tumor metastasis.^[11,12]^ Further exploration of anoikis in GC is critical to optimize individualized treatment regimens.

Therefore, we established a prognostic scoring model based on Anikis-related genes (ANRGs) and further investigated the relationship between ANRGs and GC patient prognosis, immune microenvironment, chemotherapeutic drug sensitivity, and small molecule compounds under this risk score. We developed a new feature based on ANRG, which can better predict the prognosis of GC patients. We screened more curative chemotherapy drugs and small molecule compounds to improve the personalized treatment of GC patients.

## 2. Material and methods

### 2.1. Data collection

The complete gene expression data, clinical information, and mutation data of 407 GC samples were downloaded from the TCGA online database (https://portal.gdc.cancer.gov/) (including 375 GC samples and 32 normal samples). A total of 358 ANRGs were downloaded from the GeneCard database^[13]^ (https://www.genecards.org/) and the Harmonizome portal^[14]^ (https://maayanlab.cloud/Harmonizome/). We performed univariate COX regression analysis in R software using the “survival” package to screen for ANRGs associated with prognosis (*P* value = .01).

## 3. Least absolute shrinkage and selection operator (LASSO) regression analysis

The GC samples were randomly divided into experimental and validation groups, followed by the LASSO Cox regression analysis to construct a prognostic gene signature with R package “glmnet. “The main idea of LASSO is to construct a penalty function to narrow the regression coefficient of each variable to a certain range. The risk score for each sample was calculated by a combination of weighted regression coefficients on gene expression (risk score = ∑(ð × Exp), where ð is the corresponding regression coefficient, and Exp represents the expression value of each mRNA), and based on the median GC samples were divided into high-risk and low-risk groups. The survival curves of different risk models were plotted using “survival,” and the time-dependent receiver operating characteristic (ROC) curve was plotted. The area under the ROC curve (AUC) was also calculated with the R package “timeROC.” Univariate and multivariate Cox regression analyses were used to clarify the model’s correlations among age, stage, grade, T (tumor), M (metastasis), and risk score. The statistical significance level was set at *P* < .05.

## 4. Prognostic features and assessment of the tumor immune microenvironment

CIBERSORT quantifies the abundance of specific types of immune cells in tissues or blood based on standardized gene expression data. The results of CIBERSORT have been validated by fluorescence-activated cell sorting.^[15]^ CIBERSORT and ssGSEA R scripts were used to quantify the relative proportion of infiltrating immune cells.^[15]^ We used CIBERSORT to evaluate immune cells in all GC samples and compared the differences between high-risk and low-risk samples.

## 5. Gene ontology (GO) and Kyoto encyclopedia of genes and genomes (KEGG) enrichment analysis

To explore potential signaling pathways and biological function differences between high-risk and low-risk samples, we screened differentially expressed genes (DEGs) (|log2FC| > 1.0 and FDR < 0.05) using the “limma” package. The “clusterProfiler” package was used to perform the KEGG and GO analysis of coexpressed genes. GO is a term used to describe the characteristics of genes and their products, which are divided into 3 categories: cellular components, molecular functions, and biological processes. Among them, Cellular component is used to describe the position of the gene product in the cell, Molecular function is used to describe the gene product’s function, and Biological process is used to describe the orderly biological process occurring in the cell. As a reference knowledge base, KEGG is often used to predict the role network of gene sets.

## 6. Tumor mutation burden (TMB) estimate

TMB is the mutation density of tumor genes and generally refers to the average number of mutations in the tumor genome.^[16]^ In R software, survival analysis was performed using the “survival” package combined with risk scores to study the prognostic value of TMB and risk scores in GC.

## 7. Chemotherapy response and small molecule drug screening

The Genomics of Drug Sensitivity in Cancer Project (https://www.cancerrxgene.org/) is a database used to analyze the sensitivity of anticancer drugs, which can help us predict the targeted response of anticancer drugs based on gene characteristics.^[17]^ Based on the Genomics of Drug Sensitivity in Cancer database, we used the “pRRophetic” R package to calculate the response to chemotherapy drugs in GC patients in different risk score groups. In addition, to find suitable compounds, we uploaded the up-regulated DEGs (log2 FC > 1 and *P* < .05) in the high-risk group samples to the CMAP database (https://clue.io/).^[18]^ Matches between these genes and small molecule compounds of CMAP were assessed by scoring. Positive scores indicate the stimulatory effect of the compound on the gene. At the same time, a negative score implies the inhibitory effect of the compound on the gene.

## 8. Molecular docking

The protein sequence and annotation information of the hub genes constituting the gene signature was obtained from the Universal Protein Resource (https://www.uniprot.org/, UniProt). Subsequently, the main protein structure of the hub gene was downloaded from the Protein Data Bank (http://www.rcsb.org, PDB). Chem3D software (Version 15.1) helps us convert the structure of compounds from 2D to 3D. AutoDock Tools software (Version 1.5.7) and Pymol software (http://www.pymol.org, The PyMOL Molecular Graphics System) were used to complete molecular docking and visualize the results.

## 9. Data analysis

R software (version 4.1.3, https://www.r-project.org/) and associated R packages were used to perform all graphical and statistical analyses. The *t* test was used to compare the differences between the 2 groups of samples. Survival analysis was performed using the log-rank test. *P* < .05 was considered statistically significant.

## 10. Results

### 10.1. Identification of ANRGs in GC

**Figure 1.**
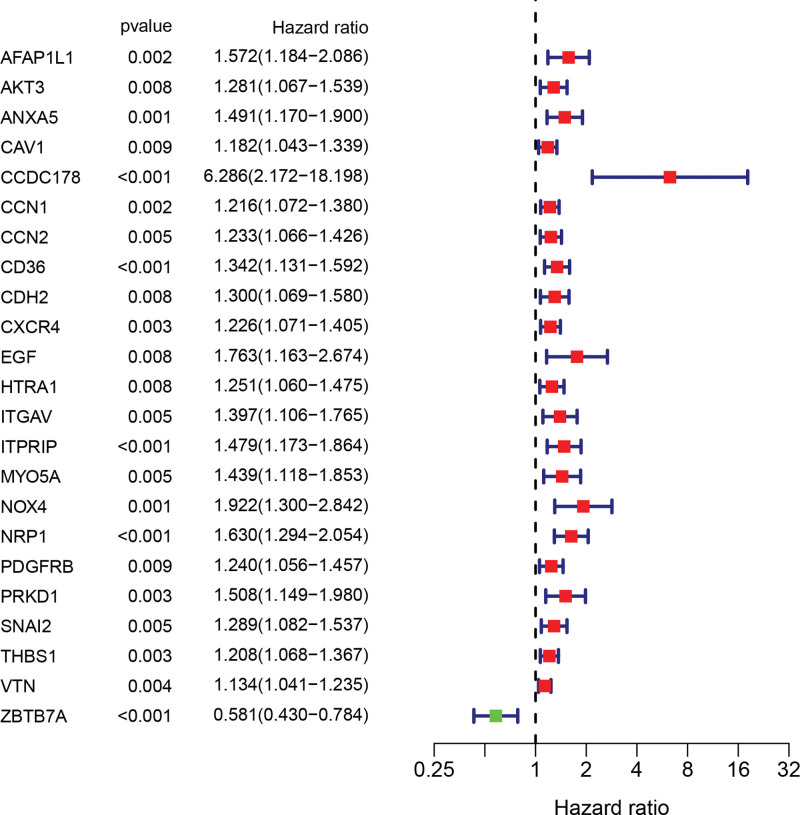
Univariate regression analysis screened out 23 prognostic-related ANRGs. According to the Hazard Ratio (HR), it can be seen that 22 genes are risk factors (HR > 1), and 1 gene is a favorable factor (HR < 1). ANRGs = Anikis-related genes.

To search for ANRGs that were meaningful in GC samples, we screened out 23 ANRGs associated with patient prognosis (*P* < .05) using univariate COX regression analysis (Fig. 1).

## 11. Establishment of the prognostic model

**Figure 2.**
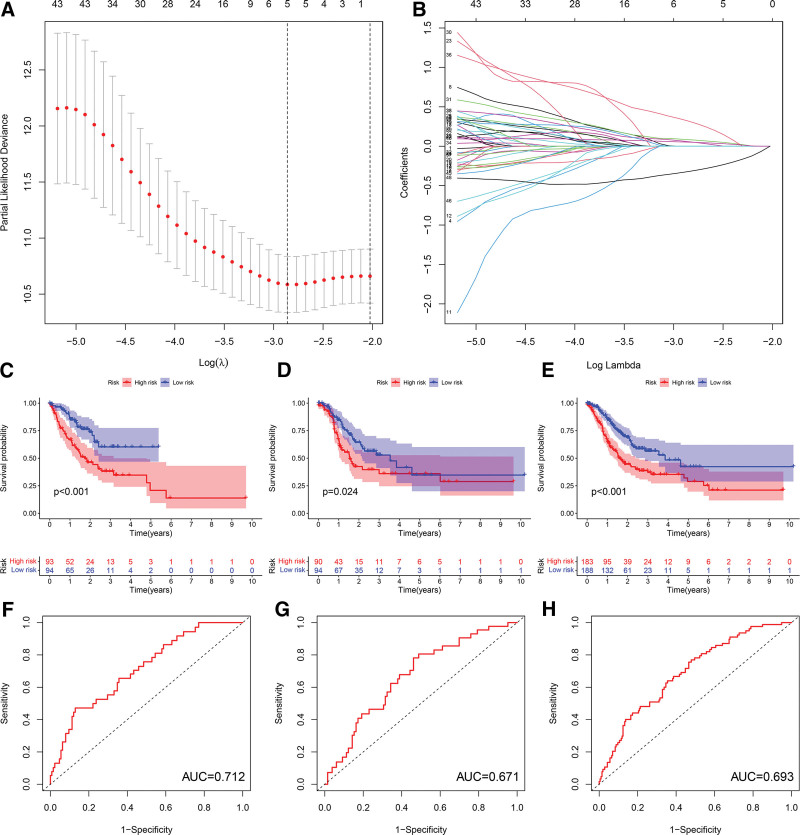
Construction and validation of the prognostic model. (A and B) LASSO regression analysis to identify optimal gene signatures. (C and E) Survival curves of high-risk and low-risk patients in the train, test, and total sample groups. (F and H) ROC curves of the prognostic models in the train, test, and total sample groups. LASSO = least absolute shrinkage and selection operator, ROC = Receiver operating characteristic curve.

Based on LASSO regression analysis, we identified a gene signature: ANXA5, CCN1, EGF, VTN, and ZBTB7A (Fig. 2A and B). The risk score of each GC sample was calculated as score = 0.059 * ANXA5 expression + 0.084 * CCN1 expression + 0.323 * EGF expression + 0.052 * VTN expression − 0.323 * ZBTB7A expression. Subsequently, we randomly divided the samples into a train group and a test group. We split each group of samples into a high-risk group and a low-risk group according to the median value of the risk score. Survival analysis showed that in the samples of the train group and the test group, the OS of the high-risk group was significantly shorter than that of the low-risk group (Fig. 2C and D). The AUC values were 0.712 and 0.671, respectively, which showed that the prognostic model had high accuracy (Fig. 2F and G). Consistently, the OS of the high-risk group in the total sample was significantly shorter than that of the low-risk group, with an AUC value of 0.693 (Fig. 2E and H).

## 12. The ability of genetic signatures to independently predict prognosis

**Figure 3.**
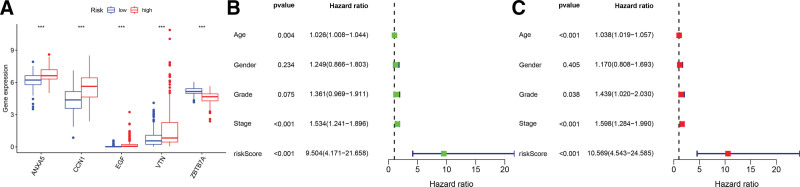
Comparison of clinicopathological features and risk score accuracy in prognosis prediction. (A) The boxplot shows the difference in the expression of the five hub genes between the high-risk and low-risk groups. (B and C) Univariate and multivariate regression analyses show gene signatures ability to predict prognosis.

In GC, we first draw boxplots to compare the expression levels of the 5 hub genes in the high-risk group and the low-risk group. It can be seen that ANXA5, CCN1, EGF, and VTN had higher expression levels in the high-risk group. At the same time, ZBTB7A was higher in the low-risk group (Fig. 3A). Univariate and multivariate COX regression analyses were performed on each variable to independently determine gene signatures’ ability to predict prognosis. In the total sample group, the results of univariate regression analysis (HR = 9.504, 95% CI = 4.171–21.658, *P* < .001) and multivariate regression analysis (HR = 10.569, 95% CI = 4.543–24.585, *P* < .001) It was shown that risk score was significantly correlated with prognosis (Fig. 3B and C). The correlation between the risk score and the clinical characteristics of the patients is summarized in Table 1. It can be seen that the samples in the high-risk group have a higher risk of metastasis (a higher proportion of M1), which is consistent with the role of ANRGs in tumors.

**Table 1:** Relationship between ANRGs and clinical characteristics of patients with gastric cancer.

Covariates	Risk	Total	High	Low	Chi	*P* value
Age	<=65	163 (43.94%)	83 (45.36%)	80 (42.55%)	0.1567	.6922
Age	>65	205 (55.26%)	99 (54.1%)	106 (56.38%)		
Age	Unknow	3 (0.81%)	1 (0.55%)	2 (1.06%)		
Gender	FEMALE	133 (35.85%)	62 (33.88%)	71 (37.77%)	0.4517	.5015
Gender	MALE	238 (64.15%)	121 (66.12%)	117 (62.23%)		
Grade	G1-2	144 (38.81%)	73 (39.89%)	71 (37.77%)	0.1323	.7161
Grade	G3	218 (58.76%)	105 (57.38%)	113 (60.11%)		
Grade	unknow	9 (2.43%)	5 (2.73%)	4 (2.13%)		
Stage	Stage I–II	161 (43.4%)	76 (41.53%)	85 (45.21%)	0.0269	.8698
Stage	Stage III–IV	187 (50.4%)	91 (49.73%)	96 (51.06%)		
Stage	Unknow	23 (6.2%)	16 (8.74%)	7 (3.72%)		
T	T1–2	96 (25.88%)	49 (26.78%)	47 (25%)	0.2793	.5972
T	T3–4	267 (71.97%)	126 (68.85%)	141 (75%)		
T	Unknow	8 (2.16%)	8 (4.37%)	0 (0%)		
M	M0	328 (88.41%)	155 (84.7%)	173 (92.02%)	4.7442	.0294
M	M1	25 (6.74%)	18 (9.84%)	7 (3.72%)		
M	Unknow	18 (4.85%)	10 (5.46%)	8 (4.26%)		
N	N0	108 (29.11%)	50 (27.32%)	58 (30.85%)	0.0433	.8352
N	N1-3	245 (66.04%)	118 (64.48%)	127 (67.55%)		
N	Unknow	18 (4.85%)	15 (8.2%)	3 (1.6%)		

ANRGs = Anikis-related genes.

## 13. The relationship between gene signature and immune microenvironment

**Figure 4.**
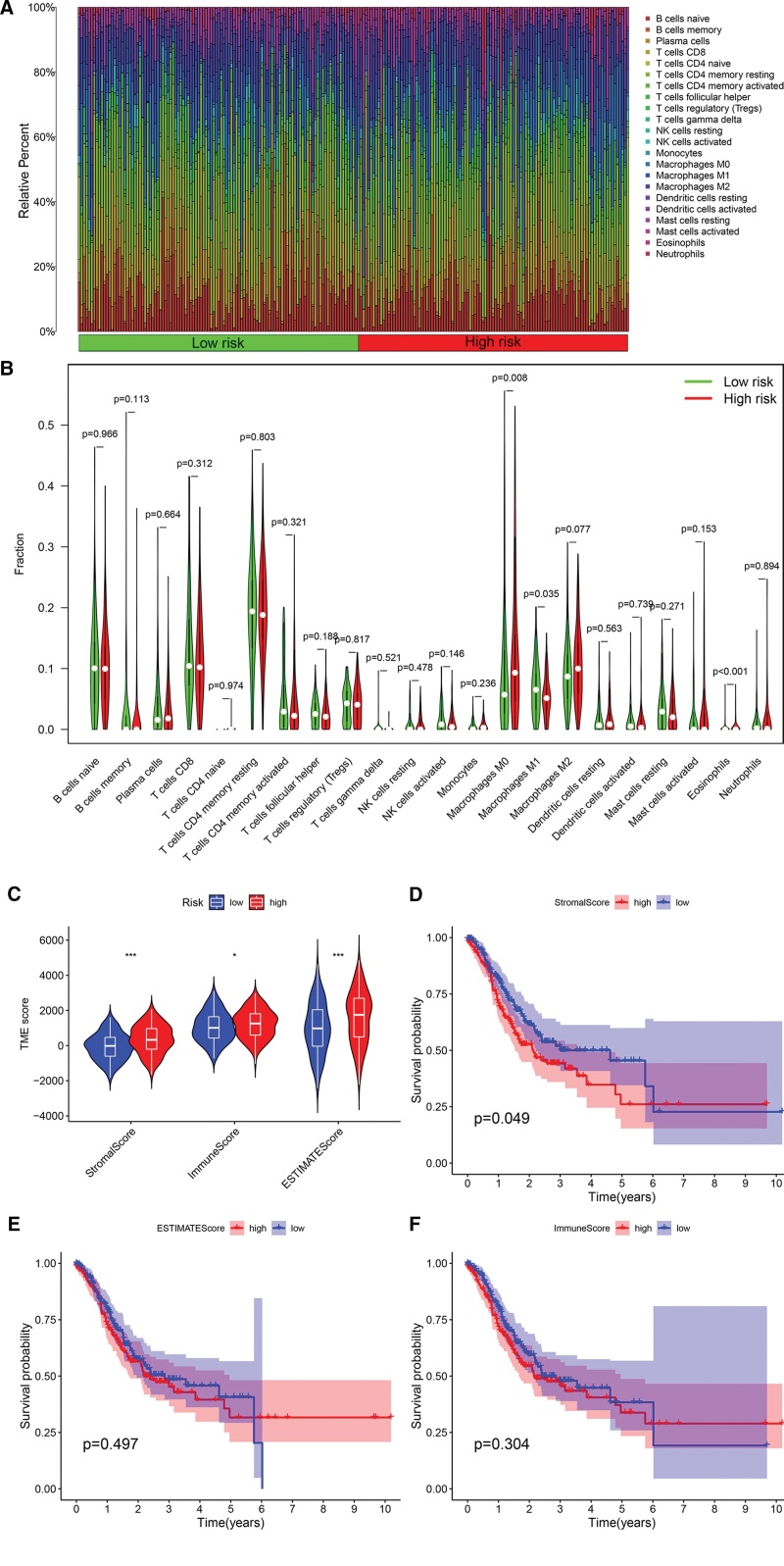
Relationship between gene signature and the immune microenvironment in GC. (A and B) There are abundant differences in 22 kinds of immune cells in the high-risk and low-risk group samples. (C) Boxplot of differences between Stromal Score, Immune Score, and ESTIMATE Score in high-risk and low-risk samples. (D–F) Survival curves of Stromal Score, Immune Score, and ESTIMATE Score in GC patients. GC = gastric cancer.

We analyzed the immune landscape in GC samples using the CIBERSORT algorithm to explore the relationship between gene signatures and the immune microenvironment. Figure 4A and B compares the abundance of 22 immune cells in the high-risk and low-risk group samples. M1 macrophages suppress solid tumor initiation, progression, metastasis, and drug resistance, while M2 macrophages have the opposite effect.^[19]^ The results of the immune analysis showed that the difference in the level of immune cell infiltration between the high-risk group and the low-risk group was mainly manifested in macrophages, and the abundance of macrophage M1 was higher in the low-risk group, which may be the reason for the better prognosis of the low-risk group one of the potential causes. Subsequently, we compared the differences in immune indicators between the high-risk and low-risk groups, including Stromal Score, Immune Score, and ESTIMATE Score. These immune indicators were significantly elevated in the high-risk group (Fig. 4C), and survival analysis in GC showed that patients with lower Stromal Score had higher prognostic performance (Fig. 4D). In contrast, Immune Score and ESTIMATE Score had no correlation with the prognosis of patients There was a clear correlation (Fig. 4E and F).

## 14. Enrichment analysis

**Figure 5.**
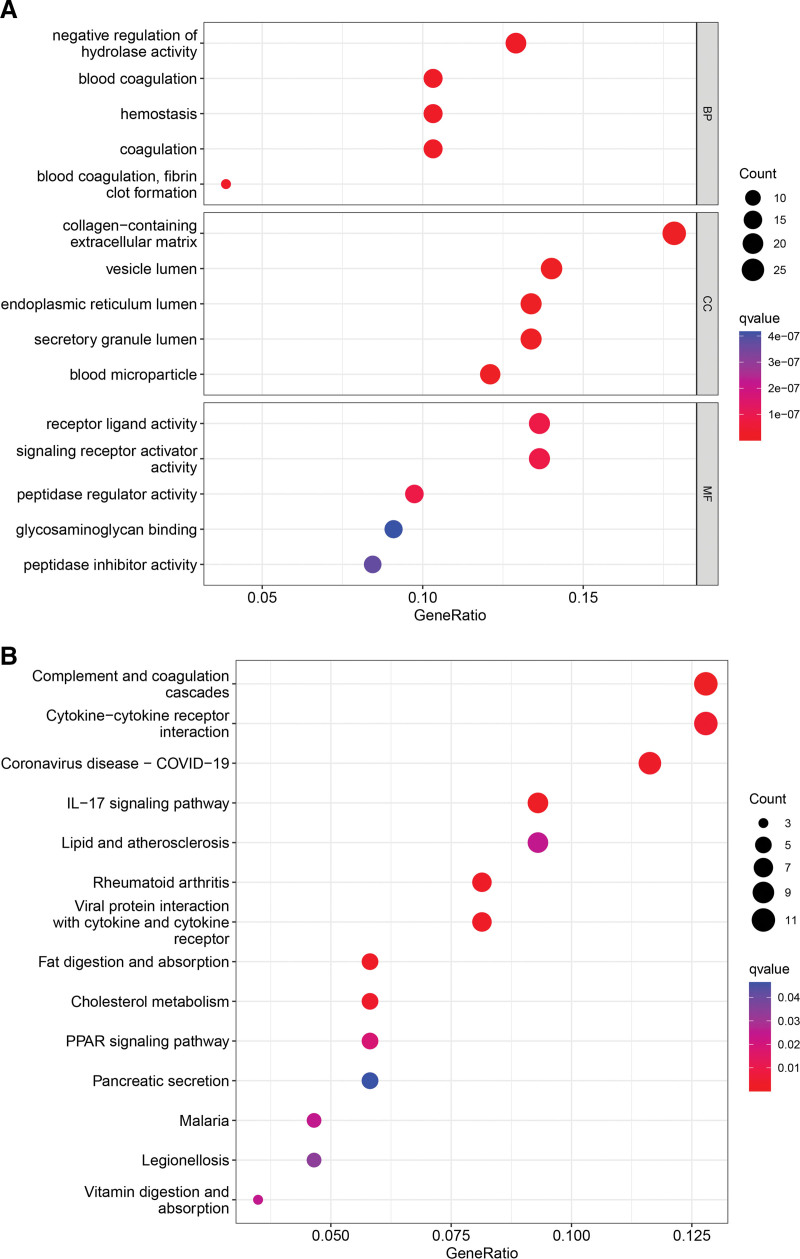
Enrichment analysis. (A and B) GO functional enrichment and KEGG pathway enrichment of differential genes in high-risk and low-risk group samples.

We performed GO and KEGG enrichment analysis on the differential genes to compare the gene expression differences between the high-risk and low-risk group samples. GO enrichment results showed that differential genes were mainly enriched in negative regulation of hydrolase activity, collagen − containing extracellular matrix, receptor-ligand activity, and signaling receptor activator activity (Fig. 5A). We found that the up-regulated DEGs in the high-risk group were mainly related to the peroxisome proliferator-activated receptor signaling pathway as well as the IL-17 signaling pathway (Fig. 5B). Peroxisome proliferator-activated receptor-γ is increased in patients with GC, which may be a molecular marker of gastritis and GC progression.^[20]^ Elevated levels of IL-17B are associated with poor prognosis in patients with GC.^[21]^

## 15. Relationship between gene signature and TMB

**Figure 6.**
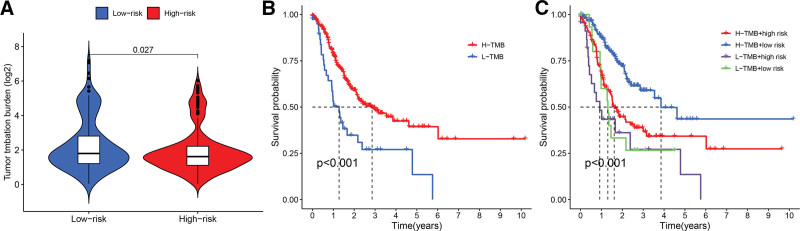
Relationship between risk score and TMB. (A) TMB differs between high-risk and low-risk groups. (B) Survival curves of TMB levels in GC patients. (C) Survival curves of TMB combined with risk score in GC patients. GO = gene ontology, GC = gastric cancer, TMB = tumor mutation burden.

TMB is used to predict the effect of immune checkpoint blockade therapy,^[22]^ so we evaluated the difference in TMB in high-risk and low-risk group samples. The results showed that GC low-risk group samples had higher TMB (Fig. 6A). Subsequent survival analysis also showed that high TMB was associated with better prognosis (Fig. 6B). After combining the risk scores, we obtained the expected results: samples with high TMB and low-risk scores had the best prognostic performance (Fig. 6C).

## 16. Chemotherapy response and small molecule drug screening

**Figure 7.**
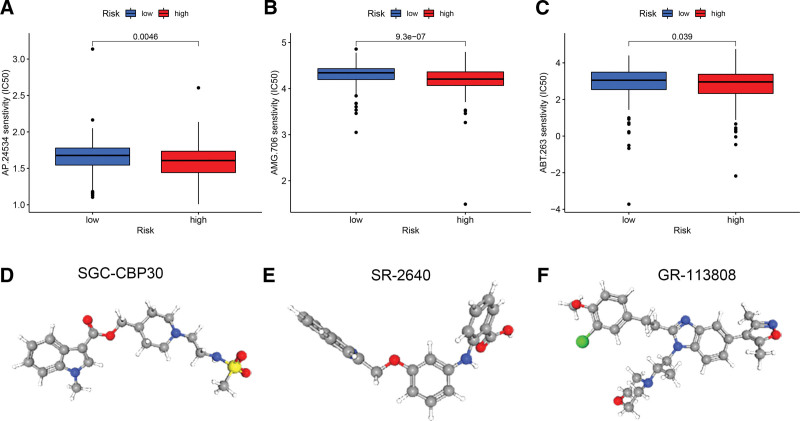
Correlation of Gene Signatures with Response to Chemotherapy. (A–C) Sensitivity analysis of patients in the high-risk and low-risk groups to three common chemotherapy drugs. (D–F) The PubChem open chemistry database predicted 3D structures of small molecule drugs, including SGC-CBP30, SR-2640, and GR-113808.

Our concern is whether the performance of gene signatures in GC patients can be fed back to the efficacy of chemotherapy drugs. Therefore, we predicted the effectiveness of commonly used chemotherapeutic agents in high-risk and low-risk samples. As shown in Figure 7A–C, the results showed that the half maximal inhibitory concentration (IC50) values of Ponatinib (ap.24534), Motesanib (amg.706), and Navitoclax (abt.263) were higher in the low-risk group (*P* < .05), indicating that patients in the high-risk group are more sensitive to chemotherapy drugs. These chemotherapy drugs have better clinical efficacy for high-risk patients. In addition, we uploaded the up-regulated 163 DEGs between the high-risk and low-risk groups to the CMap database. We screened the top 3 small molecule compounds that may help treat GC, including SGC-CBP30, SR-2640, and GR-113,808 (Fig. 7D–F).

## 17. Molecular docking analysis

**Figure 8.**
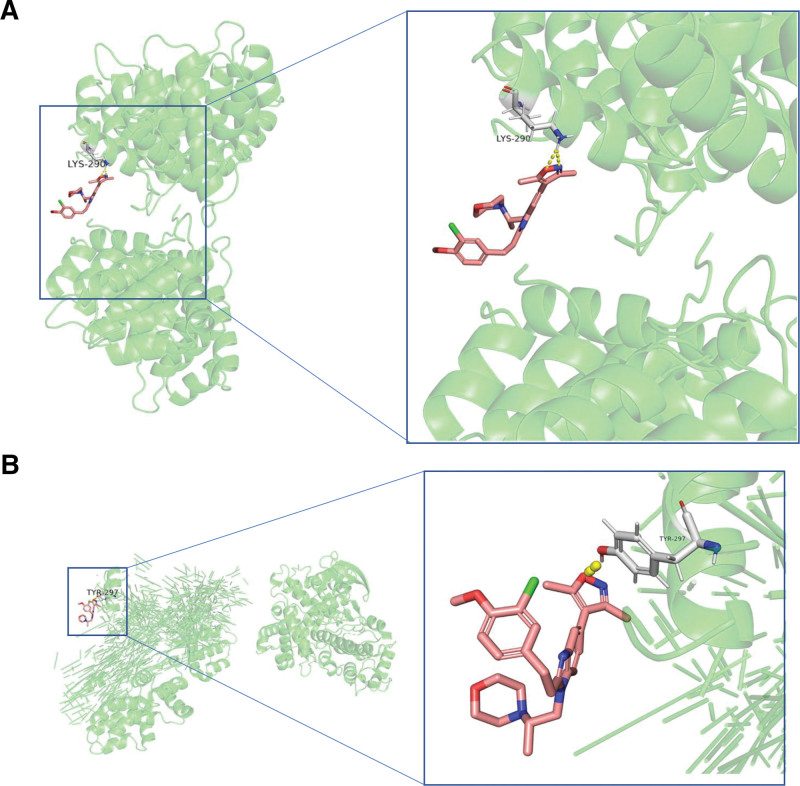
Molecular docking schema for small molecule compounds and gene signatures. (A) SGC-CBP30 binds to ANXA5 through the LYS-290 site. (B) SGC-CBP30 binds to CCN1 through the TYB-297 site.

Molecular docking helps us find the conformation of the optimal interaction between small molecule compounds and target genes for drug design and screening.^[23]^ The patient’s risk score was based on 5 hub genes, including ANXA5, CCN1, EGF, VTN, and ZBTB7A. Therefore, we used them as targets for molecular docking with the best small molecule compound SGC-CBP30 screened above. Based on energy minimization to select the best binding mode,^[24]^ we found that SGC-CBP30 can interact with ANXA5 to form hydrogen bonds through the LYS-290 site (Fig. 8A), and can also interact with CCN1 through the TYB-297 site The interactions form hydrogen bonds (Fig. 8B).

## 18. Discussion

GC is the 5th most common cancer and the third leading cause of cancer-related death worldwide.^[1]^ Surgical resection is the primary treatment for early GC. At the same time, chemotherapy is the immediate treatment for patients with advanced GC, but treatment failure is often caused by chemotherapy resistance.^[25]^ Therefore, new therapies are urgently needed to improve the cure rate of GC patients. Anikis is a specific form of programmed cell death that plays a vital role in tumor invasion and metastasis.^[26]^ Anti-Anikis-related genes are closely related to tumor invasiveness and chemotherapy resistance.^[27]^

So in this study, we screened out 5 key ANRGs signatures, including ANXA5, CCN1, EGF, VTN, and ZBTB7A, through single factor and LASSO regression analysis. We divided GC samples into high and low-risk groups based on the median of these 5 gene risk scores. In GC samples, the prognosis of the low-risk group samples was better than that of the high-risk group. Both univariate and multivariate regression analyzes showed that risk score was an independent prognostic factor for GC patients. In addition, the proportion of distant metastasis in the high-risk group was significantly higher (*P* < .05) than in the low-risk group (9.84% vs 3.72%). The above results indicate that this gene signature can be used to predict the prognosis of GC patients.

The expression of ANXA5, CCN1, EGF, and VTN was higher in the high-risk group than in the low-risk group, while the expression of ZBTB7A was lower. The expression of ANXA5 in GC was significantly increased compared with normal samples. The increased expression of ANXA5 was associated with a worse prognosis in GC patients, promoting GC by affecting extracellular matrix-related processes and immune infiltration.^[28]^ CCN1 can induce the migration and invasion of GC cells by activating the integrin/nuclear factor-κB/cyclooxygenase-2 signaling pathway,^[29–31]^ and it is also a metastatic marker of gastric cardia adenocarcinoma.^[32]^ EGF-positive GC patients have a worse prognosis than EGF-negative GC patients.^[33]^ Studies have shown that the level of VTN in serum can be used as a potential marker of prostate and endometrial cancer, promoting the progression and metastasis of cancer patients.^[34,35]^ The overexpression of ZBTB7A stops the cycle of GC cells in the S phase, promotes the apoptosis of cancer cells, and inhibits cell migration, thereby inhibiting the occurrence and development of GC.^[36]^ The above studies further confirmed our findings.

Currently, GC is mainly treated by surgery and chemotherapy. Still, since most GC patients are diagnosed at an advanced stage, traditional treatment methods are unsatisfactory.^[37]^ The tumor immune microenvironment (TME), composed of immune cells and stromal cells, is closely related to the effect of immunotherapy in cancer patients,^[38]^ and TMB can be used to predict the impact of immunotherapy.^[22]^ Therefore, in this study, we compared the differences in immune cells, TME score, and TMB between the high and low-risk groups. M1 macrophages and eosinophils in samples from the high-risk group were reduced compared to those from the low-risk group. The high-risk group’s Stromal Score, Immune Score, and ESTIMATE Score were increased in the low-risk group. The GC samples were further divided into high and low matrix score groups according to the median value of Stromal Score. The prognosis of the high Stromal Score group was worse. The TMB in the high-risk group was lower than in the low-risk group. The GC samples were further divided into high and low TMB groups according to the median value of TMB, and it was found that the high TMB group had a better prognosis. Studies have shown that M1 macrophages can inhibit tumor growth,^[39]^ and in addition, they can activate macrophage-mediated inflammatory responses to enhance the antitumor activity of paclitaxel in breast cancer patients.^[40]^ Eosinophils have antitumor effects in GC.^[41]^ This is consistent with our research results. Patients with primary GC with a high stromal score have poor overall survival, and the stromal score can be used as an independent prognostic factor for primary GC.^[42]^ In GC patients, the overall survival time of GC patients with high TMB was longer than that of GC patients with low TMB.^[43]^ This reveals the relationship between this gene signature and the immune cells, TME score, and TMB of GC patients.

Chemotherapy is the most widely used and effective method in cancer treatment, which can inhibit or kill tumor cells and improve the prognosis of patients.^[44]^ However, the emergence of chemotherapeutic drug resistance makes the treatment of tumors face significant challenges.^[45]^ Therefore, it is urgent to study the mechanism of drug resistance and improve drug sensitivity in tumor treatment.^[46]^ In this study, we identified 3 common chemotherapy drugs with statistically significant differences in IC50 between high-risk and low-risk groups, namely ponatinib (AP.24534), Bcl-2 and Bcl-xL inhibitors (ABT.263) and Motshani (AMG.706). Mutations and amplifications of receptor tyrosine kinases have been identified as drivers of GC.^[47]^ Ponatinib (AP.24534) is an oral multi-target tyrosine kinase inhibitor (TKIs),^[48]^ which can also effectively inhibit the activities of 4 FGFR kinases in vitro.^[48]^ However, resistance to AP.24534 always occurs within 1 to 2 years.^[49]^ Studies have shown that in GC TKIs-resistant cells, the level of Bcl-2 or Bcl-xL increases, and the combination of TKIs and ABT.263 can observe the apoptosis and death of GC TKIs-resistant cells, which shows that Bcl-2 and Bcl-xL mediate resistance of GC to receptor tyrosine kinases targeted therapy.^[50]^ Gastric Motesanib (AMG.706) is an oral multikinase inhibitor that can selectively inhibit vascular endothelial growth factor, platelet-derived growth factor, and kit receptors and can effectively inhibit angiogenesis and thereby inhibit malignant tumors.^[51]^ AMG.706 is well tolerated by patients, and has shown good pharmacokinetics and antitumor activity in patients with advanced refractory solid tumors, including GC.^[52]^ Therefore, predicting the chemotherapy response of GC based on the prognostic signature can be used to guide the clinical application of GC patients.

In addition, we also screened 3 targeted small molecule compounds with the most protein binding sites corresponding to up-regulated differential genes in the high-risk group, SGC-CBP30, SR-2640, and GR-113,808. Then we selected the small molecular compound with the most apparent inhibitory effect, SGC-CBP30, for molecular docking with 5 hub genes. The results showed that it could specifically bind to the LYS-290 site on the ANXA5 protein and the TYB-297 site on the CCN1 protein sex to form hydrogen bonds. The CBP/p300 inhibitor SGC-CBP30 can inhibit the proliferation of lung cancer cells and promote the apoptosis of lung cancer cells by reducing the expression level of uridine phosphorylase 1.^[53]^ Furthermore, SGC-CBP30 inhibits oncogenic KRAS and enhances the therapeutic effect of immune checkpoint inhibitors in patients with pancreatic cancer.^[54]^ The LTD4/LTE4 antagonist SR-2640 (2-[3-(2-(2-quinolylmethoxy)anilino]benzoic acid) attenuates ulcerative properties by reducing inhibition of LTB4-directed chemotaxis of neutrophils Colitis.^[55]^ The specific 5-HT4 receptor antagonist GR-113,808 inhibited the secretion of catecholamines and granulin-derived peptides from pheochromocytoma cells.^[56]^

These studies help us better understand the metastasis mechanism of gastric cancer, and ANRGs are a promising therapeutic direction for tumor metastasis. We hope that the prediction of chemotherapy drugs can help the personalized treatment of gastric cancer patients, so as to prolong the prognosis time and improve the quality of life of patients. While this study has provided valuable insights into the data analyzed, it is important to note its limitations. One major limitation is the lack of experimental verification. Secondly, our method of screening gene sets is LASSO regression analysis, and gastric cancer patients are divided into high-risk group and low-risk group by the median value of risk score for comparison. This method of analysis and grouping is the method used by most articles, but we learned that there are better clustering methods to help us classify samples.^[57]^ The 3-center relationship index proposed by this study performs best in finding the correct number of clusters and has excellent stability.^[57]^

## 19. Conclusion

Our study screened out hub genes based on ANRGs and constructed a new gene signature to predict the prognosis of GC patients and the relationship between immunity and TMB. Subsequently, we also identified chemotherapeutic drugs that can guide GC treatment and screened out the binding affinity between specific targeted drugs and specific protein sites, which provided new insights for the precise treatment of GC patients. However, our research has some limitations. Further molecular biology experiments still need to verify the relationship between the gene signature of ANRGs and the mechanism of GC transfer.

## Acknowledgments

We are very grateful to the TCGA database, which allowed us to obtain data on gastric cancer and perform new analyses. All authors reviewed the manuscript.

## Author contributions

**Conceptualization:** Yeqing Zhu.

**Data curation:** Zhijing Zhang.

**Formal analysis:** Zhijing Zhang.

**Software:** Zhijing Zhang.

**Supervision:** Yeqing Zhu.

**Writing – original draft:** Zhijing Zhang.

**Writing – review & editing:** Yeqing Zhu.
